# Methyl 4,5-diacet­oxy-1-oxo-2-phenyl­perhydro-4,6-epoxy­cyclo­penta­[*c*]pyridine-7-carboxyl­ate ethanol solvate

**DOI:** 10.1107/S1600536809045528

**Published:** 2009-11-04

**Authors:** Atash V. Gurbanov, Eugeniya V. Nikitina, Inga K. Airiyan, Vladimir P. Zaytsev, Victor N. Khrustalev

**Affiliations:** a Baku State University, Z. Khalilov St 23, Baku, AZ-1148, Azerbaijan; b Organic Chemistry Department, Russian Peoples Friendship University, Miklukho-Maklaya St 6, Moscow 117198, Russian Federation; c X-Ray Structural Centre, A. N. Nesmeyanov Institute of Organoelement Compounds, Russian Academy of Sciences, 28 Vavilov St B-334, Moscow 119991, Russian Federation

## Abstract

The title compound, the product of an acid-catalysed Wagner–Meerwein skeletal rearrangement, crystallizes as an ethanol monosolvate, C_20_H_21_NO_8_·C_2_H_6_O. The title mol­ecule comprises a fused tricyclic system containing two five-membered rings (cyclo­pentane and tetra­hydro­furan) in the usual envelope conformations and one six-membered ring (piperidinone) adopting a flattened twist–boat conformation.

## Related literature

For general background, see: Popp & McEwen (1958 ▶); Hogeveen & Van Krutchten (1979 ▶); Hanson (1991 ▶). For related structures, see: Lindberg (1980 ▶); Jung & Street (1985 ▶); Keay *et al.* (1989 ▶); Zubkov *et al.* (2004 ▶).
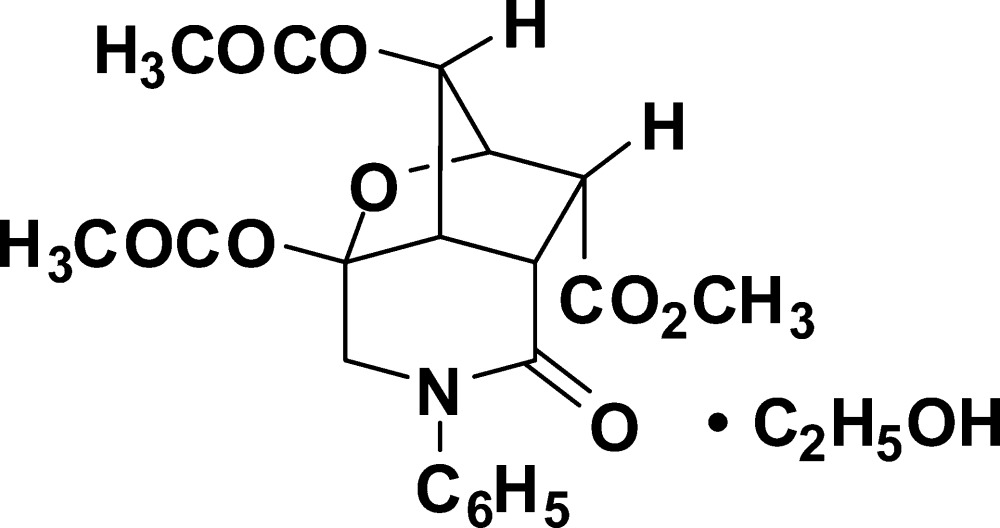


## Experimental

### 

#### Crystal data

C_20_H_21_NO_8_·C_2_H_6_O
*M* *_r_* = 449.45
Monoclinic, 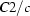

*a* = 23.2211 (13) Å
*b* = 14.9519 (8) Å
*c* = 12.9201 (7) Å
β = 107.735 (1)°
*V* = 4272.7 (4) Å^3^
*Z* = 8
Mo *K*α radiation
μ = 0.11 mm^−1^
*T* = 100 K
0.25 × 0.18 × 0.10 mm

#### Data collection

Bruker APEXII CCD diffractometer
Absorption correction: multi-scan (*SADABS*; Sheldrick, 2003 ▶) *T*_min_ = 0.976, *T*_max_ = 0.989
26806 measured reflections
6173 independent reflections
5073 reflections with *I* > 2σ(*I*)
*R*_int_ = 0.034

#### Refinement

*R*[*F* ^2^ > 2σ(*F* ^2^)] = 0.037
*wR*(*F* ^2^) = 0.097
*S* = 1.00
6173 reflections
293 parameters
H-atom parameters constrained
Δρ_max_ = 0.39 e Å^−3^
Δρ_min_ = −0.26 e Å^−3^

### 

Data collection: *APEX2* (Bruker, 2005 ▶); cell refinement: *SAINT-Plus* (Bruker, 2001 ▶); data reduction: *SAINT-Plus*; program(s) used to solve structure: *SHELXTL* (Sheldrick, 2008 ▶); program(s) used to refine structure: *SHELXTL*; molecular graphics: *SHELXTL*; software used to prepare material for publication: *SHELXTL*.

## Supplementary Material

Crystal structure: contains datablocks global, I. DOI: 10.1107/S1600536809045528/rk2176sup1.cif

Structure factors: contains datablocks I. DOI: 10.1107/S1600536809045528/rk2176Isup2.hkl

Additional supplementary materials:  crystallographic information; 3D view; checkCIF report

## Figures and Tables

**Table 1:** Hydrogen-bond geometry (Å, °)

*D*—H⋯*A*	*D*—H	H⋯*A*	*D*⋯*A*	*D*—H⋯*A*
O9—H9*O*⋯O1^i^	0.91	1.87	2.7628 (12)	168

Symmetry code: (i) 
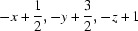
.

**Fig. 1.**
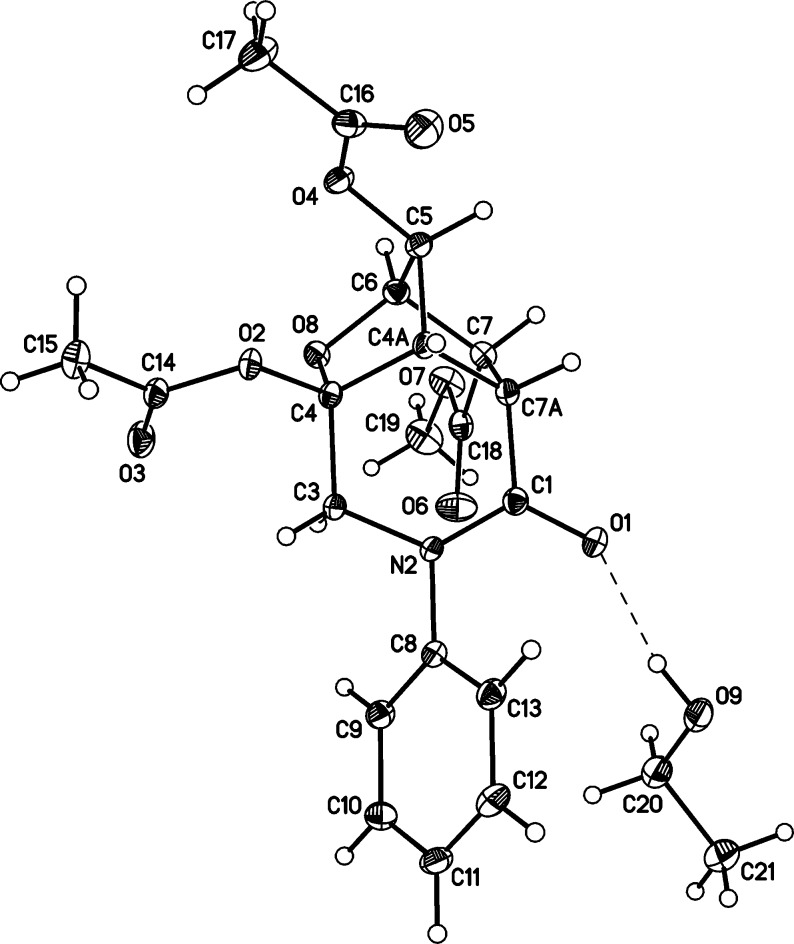
Molecular structure of I with the atom–numbering scheme. Displacement ellipsoids are shown at the 50% probability level. H atoms are presented as a small spheres of arbitrary radius. Dashed line indicates the intermolecular hydrogen bond.

**Fig. 2.**
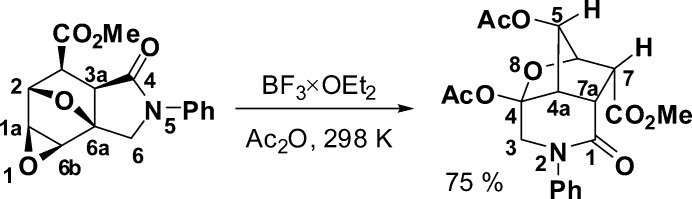
Wagner–Meerwein skeletal rearrangement of 3a,6;4,5–diepoxyisoindol–1–one.

## supplementary crystallographic information

### Comment

Wagner–Meerwein rearrangement and its analogues are largely used in current organic chemistry for the synthesis of wide range of natural derivatives, particular, terpenes and steroids. However, only a few examples of this skeletal rearrangement for nitrogen-containing compounds have been studied (Lindberg, 1980; Jung & Street, 1985; Keay *et al.*, 1989; Zubkov *et al.*, 2004). This work reports the structural characterization of a product of the acid–catalyzed Wagner–Meerwein skeletal rearrangement - methyl 1–oxo–2–phenyloctahydro–1*H*–4,6–epoxycyclopenta[*c*] pyridine–7–carboxylate (I).

Compound I crystallizes as an ethanol solvate in 1:1 ratio, *i.e.*, C_20_H_21_NO_8_^.^C_2_H_5_OH. The molecule I comprises a fused tricyclic system containing two five–membered rings (cyclopentane and tetrahydrofuran) and one six–memberedring (tetrahydropyridinone) (Fig. 1). Both five–membered rings of the bicyclic fragment have usual *envelope*–conformations, and the central six–membered ring adopts the flattened *twist–boat*–conformation. The nitrogen N2 atom has a trigonal–planar geometry (sum of the bond angles is 357.5°), which is slightly pyramidalized due to the steric reasons. The dihedral angle between the planes of the tetrahydropyridinone and phenyl rings is 57.73 (4)°. The two carboxylate substituents at the C4 and C5 carbon atoms are in the sterically unfavorable *syn*–periplanar configuration relative to the tetrahydrofuran ring. Such a disposition is explained by the direction of the Wagner–Meerwein rearrangement.

The molecules I are diastereomers and possess six asymmetric centers at the C4, C4A, C5, C6, C7 and C7A carbon atoms. The crystal of (I) is racemate and consists of enantiomeric pairs with the relative configuration of the centers *rac*-4*R**,4a*R**,5*R**, 6*S**,7*S**,7a*R**.

The ethanol solvate molecule is bound to the molecule I by the strong hydrogen bond O9—H9O···O1^i^ [O9···O1^i^ = 2.763 (1)Å, H9O···O1^i^ = 1.87Å, O9—H9O···O1^i^ = 168°]. Symmetry code: (i) -*x*+1/2, -*y*+3/2, -*z*+1.

### Experimental

An etherate of boron trifluoride (0.4 ml, 3.2 mmol) was added to a solution of methyl ether of (1a*R**,2*R**,3*R**,3a*S**, 6a*R**,6b*R**)–4–oxo–5–phenylocta–hydro–2, 6a–epoxyoxireno[*e*]isoindol–3–carboxylic acid (1.6 mmol) in 15 ml acetic anhydride. The mixture was stirred for 2 h at 293 K, diluted with 100 ml water, treated with a saturated solution of sodium carbonate and extracted by chloroform (3× 50 ml). The extract was dried by magnesium sulfate, separated and then evaporated to give white crystals of (I) (Fig. 2). Yield is 75%. M.p. = 463–464 K. IR, ν/cm^-1^: 1665, 1738 (NCO, CO_2_*Me*, CO*Me*). Mass spectrum, *m/z* (I_r_(%)): 403 [*M*+] (1), 343 (5), 256 (4), 230 (5), 188 (16), 168 (6), 124 (20), 104 (17), 77 (22), 43 (100). ^1^H NMR (CDCl_3_, 293 K): δ = 7.39 (m, 4H, H9, H10, H12, H13), 7.28 (m, 1H, H11), 4.90 (d, 1H, H5, J_5,4A_ = 1.3), 4.84 (s, 1H, H6), 4.47 (d, 1H, H3A, J_3A,3B_ = 13.4), 4.01 (d, 1H, H3B, J_3A,3B_ = 13.4), 3.73 (s, 3H, CO_2_*Me*), 3.65 (m, 1H, H4A), 3.29 (d, 1H, H7A, J_7,7A_ = 11.4), 3.28 (d, 1H, H7, J_7,7A_ = 11.4), 2.11 (s, 3H, CO*Me*), 2.04 (s, 3H, CO*Me*). ^13^C NMR (CDCl_3_, 293 K): δ = 170.1 (C1), 168.8 (CO_2_*Me*), 168.3, 166.9 (OCO*Me*), 141.5 (C8), 129.4 (C10(C12)), 127.5 (C11), 126.7 (C9(C13)), 104.5 (C4), 82.2 (C6), 76.6 (C5), 57.6 (C3), 52.5 (CO_2_*Me*), 46.2 (C7), 44.8 (C4A), 39.0 (C7A), 21.7, 20.8 (OCO*Me*).

### Refinement

The hydroxy–H atom of the ethanol solvate molecule was localized in the difference-Fourier map and included in the refinement with fixed positional and isotropic displacement parameters [*U*_iso_(H) = 1.5*U*_eq_(O)]. The other hydrogen atoms were placed in calculated positions with C—H = 0.95–1.00Å and refined in the riding model with fixed isotropic displacement parameters [*U*_iso_(H) = 1.5*U*_eq_(C) for CH_3_–groups and *U*_iso_(H) = 1.2*U*_eq_(C) for the other groups].

62 reflections, with experimentally observed *F*^2 ^deviating significantly from the theoretically calculated *F*^2^, were omitted from the refinement.

### Crystal data

**Table:**

C_20_H_21_NO_8_·C_2_H_6_O	*F*(000) = 1904
*M**_r_* = 449.45	*D*_x_ = 1.397 Mg m^−^^3^
Monoclinic, *C*2/*c*	Mo *K*α radiation, λ = 0.71073 Å
Hall symbol: -C 2yc	Cell parameters from 7315 reflections
*a* = 23.2211 (13) Å	θ = 2.5–32.3°
*b* = 14.9519 (8) Å	µ = 0.11 mm^−^^1^
*c* = 12.9201 (7) Å	*T* = 100 K
β = 107.735 (1)°	Prism, colourless
*V* = 4272.7 (4) Å^3^	0.25 × 0.18 × 0.10 mm
*Z* = 8	

### Data collection

**Table:**

Bruker APEXII CCD diffractometer	6173 independent reflections
Radiation source: Fine–focus sealed tube	5073 reflections with *I* > 2σ(*I*)
Graphite	*R*_int_ = 0.034
φ and ω scans	θ_max_ = 30.0°, θ_min_ = 1.6°
Absorption correction: multi-scan (*SADABS*; Sheldrick, 2003)	*h* = −32→32
*T*_min_ = 0.976, *T*_max_ = 0.989	*k* = −20→21
26806 measured reflections	*l* = −18→18

### Refinement

**Table:**

Refinement on *F*^2^	Primary atom site location: Direct
Least-squares matrix: Full	Secondary atom site location: Difmap
*R*[*F*^2^ > 2σ(*F*^2^)] = 0.037	Hydrogen site location: Difmap
*wR*(*F*^2^) = 0.097	H-atom parameters constrained
*S* = 1.00	*w* = 1/[σ^2^(*F*_o_^2^) + (0.05*P*)^2^ + 2.250*P*] where *P* = (*F*_o_^2^ + 2*F*_c_^2^)/3
6173 reflections	(Δ/σ)_max_ = 0.001
293 parameters	Δρ_max_ = 0.39 e Å^−^^3^
0 restraints	Δρ_min_ = −0.26 e Å^−^^3^

### Special details

**Table:**

Geometry. All s.u.'s (except the s.u. in the dihedral angle between two l.s. planes) are estimated using the full covariance matrix. The cell s.u.'s are taken into account individually in the estimation of s.u.'s in distances, angles and torsion angles; correlations between s.u.'s in cell parameters are only used when they are defined by crystal symmetry. An approximate (isotropic) treatment of cell s.u.'s is used for estimating s.u.'s involving l.s. planes.
Refinement. Refinement of *F*^2^ against ALL reflections. The weighted *R*–factor *wR* and goodness of fit *S* are based on *F*^2^, conventional *R*–factors *R* are based on *F*, with *F* set to zero for negative *F*^2^. The threshold expression of *F*^2^ > σ(*F*^2^) is used only for calculating *R*–factors(gt) *etc*. and is not relevant to the choice of reflections for refinement. *R*–factors based on *F*^2^ are statistically about twice as large as those based on *F*, and *R*–factors based on ALL data will be even larger.

### Fractional atomic coordinates and isotropic or equivalent isotropic displacement parameters (Å^2^)

**Table:**

	*x*	*y*	*z*	*U*_iso_*/*U*_eq_	
O1	0.20094 (3)	0.65247 (5)	0.44248 (6)	0.01914 (16)	
O2	0.36476 (3)	0.48946 (5)	0.28968 (6)	0.01356 (14)	
O3	0.31626 (4)	0.45288 (6)	0.11528 (6)	0.02113 (17)	
O4	0.44084 (3)	0.64234 (5)	0.34858 (6)	0.01609 (15)	
O5	0.48418 (4)	0.61959 (6)	0.52738 (7)	0.02546 (19)	
O6	0.18854 (4)	0.70348 (6)	0.19818 (7)	0.02429 (18)	
O7	0.23968 (4)	0.81107 (5)	0.14300 (6)	0.02182 (17)	
O8	0.32310 (3)	0.62521 (5)	0.20762 (5)	0.01330 (14)	
C1	0.23525 (4)	0.62345 (7)	0.39442 (8)	0.01334 (18)	
N2	0.22728 (4)	0.54326 (6)	0.34251 (7)	0.01240 (16)	
C3	0.25757 (4)	0.52025 (7)	0.26168 (8)	0.01347 (18)	
H3A	0.2327	0.5421	0.1896	0.016*	
H3B	0.2602	0.4543	0.2574	0.016*	
C4	0.32150 (4)	0.55971 (6)	0.28697 (7)	0.01159 (18)	
C4A	0.34447 (4)	0.60924 (7)	0.39505 (8)	0.01199 (17)	
H4A	0.3614	0.5708	0.4607	0.014*	
C5	0.38847 (4)	0.67743 (7)	0.37165 (8)	0.01411 (18)	
H5	0.3991	0.7262	0.4272	0.017*	
C6	0.34318 (4)	0.70795 (7)	0.26634 (8)	0.01408 (18)	
H6	0.3592	0.7533	0.2252	0.017*	
C7	0.29088 (4)	0.74178 (7)	0.30629 (8)	0.01415 (18)	
H7	0.3012	0.8026	0.3390	0.017*	
C7A	0.29279 (4)	0.67293 (7)	0.39905 (8)	0.01278 (18)	
H7A	0.3063	0.7058	0.4698	0.015*	
C8	0.17593 (4)	0.48940 (7)	0.34079 (8)	0.01284 (18)	
C9	0.13311 (5)	0.46690 (7)	0.24318 (8)	0.01558 (19)	
H9	0.1366	0.4891	0.1764	0.019*	
C10	0.08501 (5)	0.41157 (7)	0.24403 (9)	0.0186 (2)	
H10	0.0557	0.3956	0.1776	0.022*	
C11	0.07984 (5)	0.37975 (7)	0.34176 (9)	0.0187 (2)	
H11	0.0467	0.3425	0.3420	0.022*	
C12	0.12304 (5)	0.40223 (7)	0.43944 (9)	0.0188 (2)	
H12	0.1195	0.3802	0.5062	0.023*	
C13	0.17128 (5)	0.45692 (7)	0.43901 (8)	0.0163 (2)	
H13	0.2010	0.4721	0.5054	0.020*	
C14	0.35671 (5)	0.43974 (7)	0.19797 (8)	0.01534 (19)	
C15	0.40315 (5)	0.36697 (7)	0.21549 (9)	0.0204 (2)	
H15A	0.4209	0.3673	0.1558	0.031*	
H15B	0.3839	0.3090	0.2177	0.031*	
H15C	0.4349	0.3771	0.2844	0.031*	
C16	0.48659 (5)	0.61592 (7)	0.43569 (9)	0.0175 (2)	
C17	0.53882 (5)	0.58192 (9)	0.40188 (10)	0.0243 (2)	
H17A	0.5720	0.5655	0.4665	0.036*	
H17B	0.5525	0.6287	0.3617	0.036*	
H17C	0.5261	0.5293	0.3553	0.036*	
C18	0.23336 (5)	0.74775 (7)	0.21196 (8)	0.01519 (19)	
C19	0.18882 (6)	0.82342 (9)	0.04612 (9)	0.0259 (2)	
H19A	0.1958	0.8761	0.0065	0.039*	
H19B	0.1519	0.8320	0.0666	0.039*	
H19C	0.1843	0.7704	−0.0003	0.039*	
O9	0.40693 (4)	0.86539 (6)	0.51075 (7)	0.02495 (18)	
H9O	0.3727	0.8672	0.5310	0.037*	
C20	0.45662 (5)	0.86808 (8)	0.60809 (9)	0.0233 (2)	
H20A	0.4547	0.8164	0.6549	0.028*	
H20B	0.4551	0.9237	0.6488	0.028*	
C21	0.51438 (5)	0.86493 (9)	0.57799 (11)	0.0284 (3)	
H21A	0.5490	0.8649	0.6442	0.043*	
H21B	0.5166	0.9174	0.5339	0.043*	
H21C	0.5151	0.8105	0.5362	0.043*	

### Atomic displacement parameters (Å^2^)

**Table:**

	*U*^11^	*U*^22^	*U*^33^	*U*^12^	*U*^13^	*U*^23^
O1	0.0175 (4)	0.0206 (4)	0.0229 (4)	−0.0009 (3)	0.0115 (3)	−0.0067 (3)
O2	0.0151 (3)	0.0152 (3)	0.0112 (3)	0.0032 (3)	0.0053 (3)	−0.0011 (3)
O3	0.0213 (4)	0.0273 (4)	0.0145 (4)	0.0032 (3)	0.0049 (3)	−0.0050 (3)
O4	0.0118 (3)	0.0227 (4)	0.0143 (3)	0.0001 (3)	0.0047 (3)	0.0001 (3)
O5	0.0213 (4)	0.0378 (5)	0.0159 (4)	0.0030 (3)	0.0037 (3)	0.0008 (3)
O6	0.0179 (4)	0.0269 (4)	0.0240 (4)	−0.0027 (3)	0.0004 (3)	0.0072 (3)
O7	0.0250 (4)	0.0205 (4)	0.0165 (4)	−0.0022 (3)	0.0012 (3)	0.0054 (3)
O8	0.0166 (3)	0.0142 (3)	0.0093 (3)	−0.0015 (3)	0.0042 (3)	0.0007 (2)
C1	0.0135 (4)	0.0147 (4)	0.0118 (4)	0.0008 (3)	0.0037 (3)	−0.0004 (3)
N2	0.0123 (4)	0.0152 (4)	0.0113 (4)	−0.0016 (3)	0.0060 (3)	−0.0020 (3)
C3	0.0138 (4)	0.0165 (4)	0.0116 (4)	−0.0020 (3)	0.0061 (3)	−0.0035 (3)
C4	0.0128 (4)	0.0128 (4)	0.0097 (4)	0.0011 (3)	0.0043 (3)	0.0004 (3)
C4A	0.0127 (4)	0.0145 (4)	0.0091 (4)	0.0003 (3)	0.0038 (3)	−0.0001 (3)
C5	0.0118 (4)	0.0170 (4)	0.0138 (4)	−0.0006 (3)	0.0044 (3)	−0.0010 (4)
C6	0.0145 (4)	0.0143 (4)	0.0139 (4)	−0.0020 (3)	0.0049 (4)	0.0003 (3)
C7	0.0147 (4)	0.0133 (4)	0.0139 (4)	−0.0007 (3)	0.0036 (4)	−0.0008 (3)
C7A	0.0133 (4)	0.0138 (4)	0.0114 (4)	0.0000 (3)	0.0041 (3)	−0.0017 (3)
C8	0.0124 (4)	0.0135 (4)	0.0135 (4)	0.0004 (3)	0.0053 (3)	−0.0006 (3)
C9	0.0144 (4)	0.0186 (5)	0.0137 (4)	−0.0002 (4)	0.0042 (4)	0.0002 (4)
C10	0.0135 (4)	0.0212 (5)	0.0201 (5)	−0.0019 (4)	0.0036 (4)	−0.0024 (4)
C11	0.0149 (5)	0.0172 (5)	0.0270 (5)	−0.0014 (4)	0.0106 (4)	−0.0003 (4)
C12	0.0188 (5)	0.0209 (5)	0.0199 (5)	0.0017 (4)	0.0105 (4)	0.0040 (4)
C13	0.0165 (5)	0.0203 (5)	0.0131 (4)	0.0000 (4)	0.0060 (4)	0.0005 (4)
C14	0.0177 (5)	0.0168 (5)	0.0140 (4)	−0.0004 (4)	0.0085 (4)	−0.0020 (4)
C15	0.0232 (5)	0.0196 (5)	0.0208 (5)	0.0044 (4)	0.0102 (4)	−0.0014 (4)
C16	0.0140 (4)	0.0199 (5)	0.0174 (5)	−0.0027 (4)	0.0032 (4)	−0.0002 (4)
C17	0.0157 (5)	0.0332 (6)	0.0246 (6)	0.0036 (4)	0.0072 (4)	0.0026 (5)
C18	0.0181 (5)	0.0129 (4)	0.0145 (4)	0.0027 (4)	0.0049 (4)	−0.0006 (3)
C19	0.0285 (6)	0.0264 (6)	0.0172 (5)	0.0011 (5)	−0.0011 (4)	0.0063 (4)
O9	0.0180 (4)	0.0368 (5)	0.0221 (4)	−0.0021 (3)	0.0091 (3)	−0.0093 (3)
C20	0.0207 (5)	0.0302 (6)	0.0206 (5)	−0.0027 (4)	0.0089 (4)	−0.0044 (4)
C21	0.0204 (5)	0.0359 (7)	0.0315 (6)	−0.0040 (5)	0.0117 (5)	−0.0063 (5)

### Geometric parameters (Å, °)

**Table:**

O1—C1	1.2288 (12)	C8—C9	1.3886 (14)
O2—C14	1.3625 (12)	C8—C13	1.3933 (14)
O2—C4	1.4466 (11)	C9—C10	1.3927 (14)
O3—C14	1.2044 (13)	C9—H9	0.9500
O4—C16	1.3501 (13)	C10—C11	1.3884 (16)
O4—C5	1.4366 (12)	C10—H10	0.9500
O5—C16	1.2040 (13)	C11—C12	1.3930 (16)
O6—C18	1.2001 (13)	C11—H11	0.9500
O7—C18	1.3384 (13)	C12—C13	1.3882 (15)
O7—C19	1.4475 (14)	C12—H12	0.9500
O8—C4	1.4264 (11)	C13—H13	0.9500
O8—C6	1.4518 (12)	C14—C15	1.4998 (15)
C1—N2	1.3587 (13)	C15—H15A	0.9800
C1—C7A	1.5125 (13)	C15—H15B	0.9800
N2—C8	1.4335 (12)	C15—H15C	0.9800
N2—C3	1.4668 (12)	C16—C17	1.4978 (15)
C3—C4	1.5372 (13)	C17—H17A	0.9800
C3—H3A	0.9900	C17—H17B	0.9800
C3—H3B	0.9900	C17—H17C	0.9800
C4—C4A	1.5262 (13)	C19—H19A	0.9800
C4A—C5	1.5372 (14)	C19—H19B	0.9800
C4A—C7A	1.5451 (13)	C19—H19C	0.9800
C4A—H4A	1.0000	O9—C20	1.4252 (14)
C5—C6	1.5145 (14)	O9—H9O	0.9090
C5—H5	1.0000	C20—C21	1.5067 (16)
C6—C7	1.5425 (14)	C20—H20A	0.9900
C6—H6	1.0000	C20—H20B	0.9900
C7—C18	1.5118 (14)	C21—H21A	0.9800
C7—C7A	1.5703 (14)	C21—H21B	0.9800
C7—H7	1.0000	C21—H21C	0.9800
C7A—H7A	1.0000		
			
C14—O2—C4	117.69 (8)	C8—C9—C10	119.40 (9)
C16—O4—C5	115.76 (8)	C8—C9—H9	120.3
C18—O7—C19	116.10 (9)	C10—C9—H9	120.3
C4—O8—C6	106.50 (7)	C11—C10—C9	120.16 (10)
O1—C1—N2	123.31 (9)	C11—C10—H10	119.9
O1—C1—C7A	120.55 (9)	C9—C10—H10	119.9
N2—C1—C7A	115.91 (8)	C10—C11—C12	120.22 (10)
C1—N2—C8	119.40 (8)	C10—C11—H11	119.9
C1—N2—C3	122.39 (8)	C12—C11—H11	119.9
C8—N2—C3	115.74 (8)	C13—C12—C11	119.85 (10)
N2—C3—C4	113.58 (8)	C13—C12—H12	120.1
N2—C3—H3A	108.8	C11—C12—H12	120.1
C4—C3—H3A	108.8	C12—C13—C8	119.70 (10)
N2—C3—H3B	108.8	C12—C13—H13	120.2
C4—C3—H3B	108.8	C8—C13—H13	120.2
H3A—C3—H3B	107.7	O3—C14—O2	123.08 (9)
O8—C4—O2	110.24 (7)	O3—C14—C15	125.65 (10)
O8—C4—C4A	104.24 (7)	O2—C14—C15	111.26 (9)
O2—C4—C4A	106.47 (7)	C14—C15—H15A	109.5
O8—C4—C3	110.22 (8)	C14—C15—H15B	109.5
O2—C4—C3	110.19 (8)	H15A—C15—H15B	109.5
C4A—C4—C3	115.25 (8)	C14—C15—H15C	109.5
C4—C4A—C5	102.12 (7)	H15A—C15—H15C	109.5
C4—C4A—C7A	105.73 (8)	H15B—C15—H15C	109.5
C5—C4A—C7A	99.63 (8)	O5—C16—O4	123.12 (10)
C4—C4A—H4A	115.8	O5—C16—C17	125.93 (10)
C5—C4A—H4A	115.7	O4—C16—C17	110.95 (9)
C7A—C4A—H4A	115.8	C16—C17—H17A	109.5
O4—C5—C6	108.88 (8)	C16—C17—H17B	109.5
O4—C5—C4A	117.00 (8)	H17A—C17—H17B	109.5
C6—C5—C4A	93.17 (7)	C16—C17—H17C	109.5
O4—C5—H5	112.1	H17A—C17—H17C	109.5
C6—C5—H5	112.1	H17B—C17—H17C	109.5
C4A—C5—H5	112.1	O6—C18—O7	123.87 (10)
O8—C6—C5	103.73 (8)	O6—C18—C7	126.90 (10)
O8—C6—C7	107.10 (8)	O7—C18—C7	109.22 (9)
C5—C6—C7	101.55 (8)	O7—C19—H19A	109.5
O8—C6—H6	114.4	O7—C19—H19B	109.5
C5—C6—H6	114.4	H19A—C19—H19B	109.5
C7—C6—H6	114.4	O7—C19—H19C	109.5
C18—C7—C6	109.99 (8)	H19A—C19—H19C	109.5
C18—C7—C7A	117.81 (8)	H19B—C19—H19C	109.5
C6—C7—C7A	101.44 (8)	C20—O9—H9O	106.7
C18—C7—H7	109.1	O9—C20—C21	108.45 (9)
C6—C7—H7	109.1	O9—C20—H20A	110.0
C7A—C7—H7	109.1	C21—C20—H20A	110.0
C1—C7A—C4A	112.52 (8)	O9—C20—H20B	110.0
C1—C7A—C7	118.12 (8)	C21—C20—H20B	110.0
C4A—C7A—C7	102.72 (7)	H20A—C20—H20B	108.4
C1—C7A—H7A	107.7	C20—C21—H21A	109.5
C4A—C7A—H7A	107.7	C20—C21—H21B	109.5
C7—C7A—H7A	107.7	H21A—C21—H21B	109.5
C9—C8—C13	120.67 (9)	C20—C21—H21C	109.5
C9—C8—N2	120.81 (9)	H21A—C21—H21C	109.5
C13—C8—N2	118.46 (9)	H21B—C21—H21C	109.5
			
O1—C1—N2—C8	1.28 (15)	C5—C6—C7—C7A	−36.36 (9)
C7A—C1—N2—C8	175.88 (8)	O1—C1—C7A—C4A	147.32 (9)
O1—C1—N2—C3	162.71 (9)	N2—C1—C7A—C4A	−27.44 (12)
C7A—C1—N2—C3	−22.70 (13)	O1—C1—C7A—C7	−93.22 (12)
C1—N2—C3—C4	34.80 (13)	N2—C1—C7A—C7	92.02 (11)
C8—N2—C3—C4	−163.14 (8)	C4—C4A—C7A—C1	60.66 (10)
C6—O8—C4—O2	−116.23 (8)	C5—C4A—C7A—C1	166.26 (8)
C6—O8—C4—C4A	−2.31 (9)	C4—C4A—C7A—C7	−67.41 (9)
C6—O8—C4—C3	121.92 (8)	C5—C4A—C7A—C7	38.19 (9)
C14—O2—C4—O8	−62.44 (10)	C18—C7—C7A—C1	−6.00 (13)
C14—O2—C4—C4A	−174.93 (8)	C6—C7—C7A—C1	−126.08 (9)
C14—O2—C4—C3	59.44 (10)	C18—C7—C7A—C4A	118.46 (9)
N2—C3—C4—O8	−112.96 (9)	C6—C7—C7A—C4A	−1.62 (9)
N2—C3—C4—O2	125.15 (8)	C1—N2—C8—C9	119.00 (11)
N2—C3—C4—C4A	4.66 (12)	C3—N2—C8—C9	−43.63 (13)
O8—C4—C4A—C5	−30.95 (9)	C1—N2—C8—C13	−63.70 (13)
O2—C4—C4A—C5	85.63 (8)	C3—N2—C8—C13	133.67 (10)
C3—C4—C4A—C5	−151.87 (8)	C13—C8—C9—C10	0.25 (15)
O8—C4—C4A—C7A	72.84 (9)	N2—C8—C9—C10	177.49 (9)
O2—C4—C4A—C7A	−170.59 (7)	C8—C9—C10—C11	0.39 (16)
C3—C4—C4A—C7A	−48.09 (10)	C9—C10—C11—C12	−0.65 (16)
C16—O4—C5—C6	177.19 (8)	C10—C11—C12—C13	0.26 (16)
C16—O4—C5—C4A	−78.93 (11)	C11—C12—C13—C8	0.38 (16)
C4—C4A—C5—O4	−64.12 (10)	C9—C8—C13—C12	−0.64 (15)
C7A—C4A—C5—O4	−172.64 (8)	N2—C8—C13—C12	−177.94 (9)
C4—C4A—C5—C6	48.96 (8)	C4—O2—C14—O3	1.47 (14)
C7A—C4A—C5—C6	−59.56 (8)	C4—O2—C14—C15	−177.06 (8)
C4—O8—C6—C5	35.64 (9)	C5—O4—C16—O5	1.18 (15)
C4—O8—C6—C7	−71.26 (9)	C5—O4—C16—C17	−179.20 (9)
O4—C5—C6—O8	68.37 (9)	C19—O7—C18—O6	−1.27 (15)
C4A—C5—C6—O8	−51.60 (8)	C19—O7—C18—C7	178.29 (9)
O4—C5—C6—C7	179.39 (8)	C6—C7—C18—O6	113.51 (12)
C4A—C5—C6—C7	59.43 (8)	C7A—C7—C18—O6	−1.98 (15)
O8—C6—C7—C18	−53.38 (10)	C6—C7—C18—O7	−66.03 (10)
C5—C6—C7—C18	−161.82 (8)	C7A—C7—C18—O7	178.47 (8)
O8—C6—C7—C7A	72.08 (9)		

### Hydrogen-bond geometry (Å, °)

**Table:**

*D*—H···*A*	*D*—H	H···*A*	*D*···*A*	*D*—H···*A*
O9—H9O···O1^i^	0.91	1.87	2.7628 (12)	168

Symmetry codes: (i) −*x*+1/2, −*y*+3/2, −*z*+1.
